# Control and Guidance of Low-Cost Robots via Gesture Perception for Monitoring Activities in the Home

**DOI:** 10.3390/s151229853

**Published:** 2015-12-11

**Authors:** Angel D. Sempere, Arturo Serna-Leon, Pablo Gil, Santiago Puente, Fernando Torres

**Affiliations:** Physics, Systems Engineering and Signal Theory Department, University of Alicante, San Vicente del Raspeig, E-03690 Alicante, Spain; adss1@alu.ua.es (A.D.S.); asl37@alu.ua.es (A.S.-L.); Pablo.gil@ua.es (P.G.); Fernando.torres@ua.es (F.T.)

**Keywords:** robot systems, human-robot interaction, 3D gesture perception, 3D descriptors

## Abstract

This paper describes the development of a low-cost mini-robot that is controlled by visual gestures. The prototype allows a person with disabilities to perform visual inspections indoors and in domestic spaces. Such a device could be used as the operator's eyes obviating the need for him to move about. The robot is equipped with a motorised webcam that is also controlled by visual gestures. This camera is used to monitor tasks in the home using the mini-robot while the operator remains quiet and motionless. The prototype was evaluated through several experiments testing the ability to use the mini-robot’s kinematics and communication systems to make it follow certain paths. The mini-robot can be programmed with specific orders and can be tele-operated by means of 3D hand gestures to enable the operator to perform movements and monitor tasks from a distance.

## 1. Introduction

Mobility is one of the most important facets of any person’s autonomous capabilities. However, motor disabilities constitute a major issue in our society. A significant proportion of older people have serious mobility problems. According to recent reports, approximately 20% of people aged 70 years or older and 50% of people aged 85 or over report difficulties in performing basic daily living activities [1]. Mobility problems are common and impede domestic activities. Furthermore, current demographics show that the elderly population (aged over 65) in industrialised countries is continuously increasing [2,3].

The assistance of a machine to perform autonomous tasks would be of great benefit to many people. The lack of human resources available to assist people with mobility problems has naturally led to the creation of systems for achieving autonomous mobility. Future research in this area should strive to make life easier in the home. Today, many basic household chores can be supported through technology, in the form of domestic robots. 

Several research projects exist for assisting the elderly through robotic solutions for healthcare and quality of life, including home care robots: Georgia Tech has Cody; CMU has Herb; the Fraunhofer Institute has Care-O-Bot; Yale, USC, and MIT are running an NSF-funded project on Socially Assistive Robotics; and CIR and KAIST in Korea are conducting their own robot projects [4]. The University of Reading has also been working on a project called Hector: Robotic Assistance for the Elderly [5].

However, the vast majority of the current robotic assistance solutions present a serious handicap, namely the cost of domestic robots is still prohibitive for the average family. At the same time, the emergence of single-board computers, such as the Raspberry Pi, and the popularisation of microcontroller evaluation boards, such as Arduino, offer new possibilities for realising capable robots on a significantly lower budget.

Additionally, tele-operation and control of machines such as robotic arms or vehicles with various haptic tools have been on the market for several years in the form of various commercial products [6]. One example of such a product is the ABB FlexPendant [7], a controller that combines touchscreen controls with a joystick and physical buttons, thereby allowing the user to exert direct control over a robotic arm or mixed control using pre-programmed scripts; another example is the Rovio WowWee [8], an RC car that runs on a web server that can be controlled using a computer or tablet via the Internet. Moreover, non-haptic systems have also been used commercially, though so far only for leisure products, such as those for computer interface control (Samsung Smart Interaction, an interface for controlling a television through voice commands and gestures) or video game interaction (Sony EyeToy or Microsoft Kinect). Because of these video game controllers, the popularisation of depth cameras has led to a reduction in the cost of manufacturing sensors using various underlying technologies a fact which has made these elements suitable options for recognising body parts, thus enabling interaction with computers and replacing traditional haptic interfaces. By focusing the problem of body part recognition on the identification of various hand gestures, we can allow individuals with mobility problems to interact easily with a servant robotic platform by associating commands with these gestures.

Service robots can help physically disabled people to live a more independent life and can also offer sensory support. In the near future, they may well become a common household item adapted for the home. Moreover, they could be connected to emergency services that can provide help and support 24 h a day, seven days a week [9]. A low-cost robot would be a promising solution for individuals who, due to poor memory skills or mobility are unsafe at home [10,11]. The purpose of our mobile robot is to serve as a prototype of a low-cost service robot for monitoring rooms and allowing individuals to monitor locations inside their homes. Our low-cost mobile robot prototype was designed with two main features: the ability to be remotely controlled by hand gestures captured by RGBD sensors (Kinect) and the capability of moving in an autonomously controlled manner using a Raspberry Pi microcontroller.

In recent years, numerous attempts have been made resolve the problems of hand gesture recognition using real-time depth sensors [12] and many other efforts have focused on building remote control applications for robots making use of them [13,14] due to their potential applications in contactless human-computer interaction. Thus, considerable progress has been made in this area and a number of algorithms addressing different aspects of the problem have been previously proposed. Techniques and methods to improve the pre-processing algorithms and to reduce the quantization error caused by low resolution of Kinect [15] for hand recognition include methods based on local shape detection using superpixel and colour segmentation techniques [16], approaches for hand gesture recognition using learning techniques such as PCA and multilayer perceptron on a large database of samples [17] and so forth. This paper proposes a system based on state machine that extracts accurate 3D hand gestures using a three-dimensional descriptor. The combination of 3D descriptor as VFH and the implementation of a state machine improve the results reducing the recognition error in comparison with the ground truth. Our system uses the skeleton information from Kinect to produce markless hand extraction similar to [16] however, unlike that work in that we use a global descriptor instead of local shape of superpixels to retain the overall shapes of gestures to be recognized. Also, our training phase does not require as much data and time as in [17]. Current methods are generally based on appearance or models and they are dependent on the image features, invariance properties and number of gestures to be recognized [12,18]. Moreover, they can only handle a discrete set of hand gestures if they are to run in real time. To mitigate this fact and achieve robustness, our system can handle a discrete set of hand gestures with a few simple hand gestures by combining both in order to build a sequence of two or more gestures which can be associated with different orders or commands.

This paper is organised as follows: Section 2 describes the proposed method. We begin by specifying the components of the physical platform to be tele-operated and the methodology for commanding its operations. We then describe how the perception system is designed to enable non-haptic tele-operation by using a depth sensor to locate the hands of the operator-user in space and performing gesture analysis, based on previous training. Section 3 presents several experiments that cover various aspects of the functionality and performance of the proposed method. Finally, we report the conclusions of this work.

## 2. Proposed Method 

The motivation for our work is to facilitate the performance of surveillance and verification tasks by persons with mobility issues in indoor living spaces. 

This project has two major components: the client computer and the robot. The client device displays the camera signal that is acquired by the robot and detects hand gestures using a Kinect. Kinect was chosen for two main reasons: firstly, it is necessary to have a large field of vision so that most of the operator’s body can be mapped (in contrast to other sensors like Leap Motion); and secondly, it is the cheapest and the most extensively used of its kind, a fact that allows us to easily integrate it with reusing code, from open-free libraries without platform constraints. The robot, called Charlie [19], is capable of moving and of streaming the camera signal. It also incorporates a servomotor that controls the inclination of the camera and several optical sensors that capture images of the ground for line detection. By virtue of certain pre-programmed behaviours, it is possible to activate a script to execute a task, such as going to a specific room, or to direct actions, including the positioning of the servo. The selection of the robot provides flexibility in the application design; however it can be performed with different platforms like IRobot Create 2 with small changes. The Kinect is placed in the location where the person with reduced mobility spends most of their time, thereby providing that person with full control of the robot at all times. The camera on the robot continuously streams its forward field of vision; an RGB 2D model camera is used for this purpose because it offers a useful, good-quality image for a low price. Because it is not focused on the user and its technology is less robust in several aspects (such as background changes, differences in lighting, and the presence of multiple objects on the same plane) due to the difficulty of applying descriptors to a point cloud, this sensor is not used for item recognition.

**Table 1:** The components of Charlie and their cost (September 2014).

Component	Cost in €
GoShield-GR (include board, motor driver, wheels)	114.76
Arduino Due	47.19
Raspberry Pi B	35.99
Raspberry camera module	22.95
32 GB class 10 Samsung SD card	17.48
S3003 servomotor	3.45
SR04 distance sensor	1.11
Ralink RT5370 Wi-Fi USB adapter	6.95
Cables, enclosures, 4 AA batteries	17.00
**TOTAL**	**266.88**

Another major goal is to make this technology available to people on low incomes by always striving to use cost-effective components. The total cost of the robot prototype is less than 270€, with autonomy around 1 h (Table 1). The client system can be installed on any x86-compatible computer with the addition of a Kinect.

### 2.1. Robot Design 

The robot prototype, called Charlie, was built using readily available and cost-effective components. The main components are a Raspberry Pi, which controls the high-level commands for the robot, and an Arduino Due, which is responsible for handling the commands for the low-level API. Both API levels are described in the next section.

The core system is a Raspberry Pi that runs Raspbian, a Debian-based GNU/Linux distribution. This module manages the command server and streams the camera signal through a motion JPEG server. The command server uses the WebSocket protocol. This protocol allows the client device to control the components of the robot through the API. For communication with the client device, the system has a Wi-Fi adapter and is able to create an *ad hoc* network or to connect to an existing network. 

**Figure 1:**
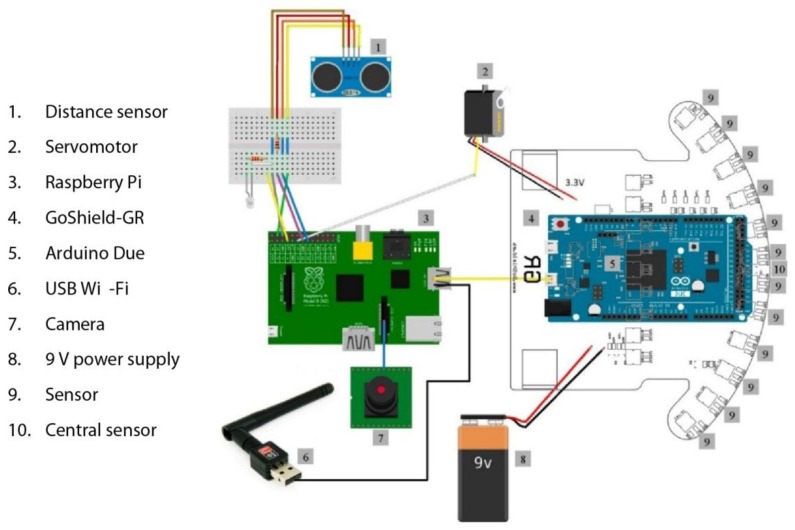
Robot design schematic.

The Arduino Due is responsible for controlling the components of the GoShield-GR shield. This microcontroller receives control commands from the Raspberry Pi via USB and outputs electrical signals to the various components of the GoShield-GR shield. Of these, the primary components are 2 DC motors for movement and 21 CNY70 optical sensors that are focused on the ground for line detection. The other components are 14 LEDs and a buzzer, for indication purposes. A schematic diagram of the robot is provided in Figure 1.

In total, the robot API consists of 21 optical sensors for detecting lines on the ground, 2 DC motors, a servomotor that controls the inclination of the camera, a distance sensor, and 14 LEDs and a buzzer to serve as indicators (Table 2). 

**Table 2:** Accessible components of the robot.

Component	Quantity
75:1 Micro Metal DC/Gearmotor HP	2
CNY70 optical sensors	21
Raspberry camera module 1080 p	1
SR04 distance sensor	1
S3003 servomotor	1
LEDs	14
Buzzer	1

### 2.2. Robot Commands API 

Charlie the robot is capable of executing pre-programmed behaviours and taking direct actions through its API. Examples of its pre-programmed behaviours include moving to a specific room in the house, performing a check of each room, or returning to the user’s room. The direct actions it can take include controlling the inclination of the camera or its own movement (such as going straight, stopping its movement or turning left) and measuring the distance to the closest object.

The API is divided into two levels: direct action commands and scripting commands. The first level controls the direct action commands (cmd). These commands are transmitted using 1 to 3 bytes. They are used to control the components of the robot. These commands are related to the kinematics of the robot, the positioning of the servomotor, and the read-out of the distance sensor or the optical sensors. The first byte of a command signal indicates the number of commands to be issued. Depending on the command, it may also have zero, one or two parameters, each of which is 1 byte in length. Most direct action commands are retransmitted without alteration by the Raspberry Pi to the Arduino Due board through USB communication. The remaining commands are implemented by the Raspberry Pi itself, which is responsible for controlling the servomotor and measuring distances.

The high-level API enables the saving, loading, deletion and execution of the scripts for the pre-programmed tasks. The scripting language is Python, and it is possible to use the full set of instructions and standard libraries of this language plus 2 additional functions that enable specific functionalities of the robot, one for the execution of commands and the other for receiving data from the hardware. The first of these functions is execute Command(cmd, [p1], [p2]) for executing direct action commands with the API. If the command returns a value (reads a sensor), then the appropriate function to use is executeReadCommand(cmd). None of the sensor reading commands has parameters. With respect to the syntax, the first byte indicates the type of command. If the command is related to the memory (*i.e*., if it saves, loads or deletes a script), then the next byte indicates the ID of the relevant script. Therefore, there are 256 available positions for storing scripts. The remainder of the command bytes contain the Python code. There is no length constraint at this API level. For saving a script, the server receives a command whose first byte contains the value “50” and whose second byte indicates the ID of the script (between 0 and 255); the following bytes contain the Python script itself. Consequently, the server will save the script in a file that is named with the specified ID. A command for executing a script that was previously saved consists of two bytes, where the first byte contains the value 51 and the second byte contains the script ID. The server loads the file that contains the script and executes it using the exec function of Python. 

### 2.3. Robot Communication 

**Figure 2:**
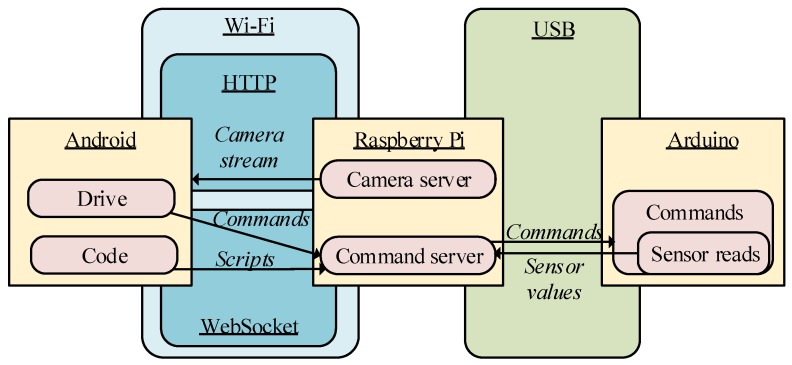
Communication scheme of the robot.

One important issue related to the robot scheme is the implementation of communication among the Android application, the Raspberry Pi and the Arduino board. This communication is performed in two steps: one between the Android application and the Raspberry Pi and the other between the Raspberry Pi and the Arduino board. The first is high-level communication; it allows the transmission of commands and scripts to the robot and the reception of the camera signal by the user of the Android application. This channel uses the IEEE 802.11 standard for Wi-Fi communication to connect through HTTP for the camera signal and uses WebSockets in the command server. The second level of communication is performed between the Raspberry Pi and the Arduino board. This channel is used for low-level communication; it uses a connection through the USB port of the Raspberry Pi to the USB port of the Arduino board, enabling serial communication between them for the transmission of commands and sensor values. The scheme of the communication channels is depicted in Figure 2.

The steps of initialising communication are as follows:

1. The Raspberry Pi creates an ad hoc Wi-Fi hotspot to connect to the Android user
2. The Raspberry Pi initialises the camera server
3. The Raspberry Pi initialises the command server
  - It opens a serial communication channel with the Arduino board
  - It starts up the WebSocket
4. The Android application connects to the Wi-Fi network of the Raspberry Pi
5. The Android application generates a WebSocket connection
6. The Raspberry Pi accepts the Android WebSocket connection
7. The Android application is sent a command by WebSockets [1,200,123] (3 bytes)
8. The Raspberry Pi receives the command
9. The Raspberry Pi translates the command and sends it to the Arduino board
10. The Arduino board translates the command to the electrical references of the motors

### 2.4. Perception System

#### 2.4.1. Human-Hand Detection Process

**Figure 3:**
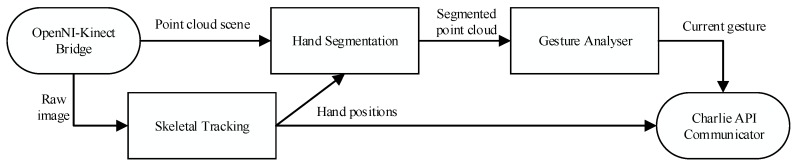
Data flow among the different software components of the application.

Hand detection has been widely discussed in the literature on perception systems for interaction between humans and electronic devices. New sensor technologies have facilitated the sensing of human body parts for remote control of avatars and robotic devices. 3D cameras such as RGBD or time-of-flight (ToF) cameras enable the extraction of human gestures and movements [20], and it is anticipated that they can be used to control devices from a distance. Master-slave architectures such as haptic devices or infrared remote control have been widely used in previous works to move robots both with feedback [21,22] and without feedback [23]. At present, visual sensors have replaced these systems in many cases because of their cost and versatility. Cameras allow the extraction of the hands of a user using a combination of several image processing techniques, such as skin colour segmentation (based on a combination of static and dynamic thresholds) [24,25], background subtraction using different scenes of a video stream [26] and shape recognition based on morphological information (*i.e*., curvature calculations and convexity defects [27]). The depth information provided by RGBD and ToF sensors helps us to solve the two main drawbacks of this approach, namely, the dependence on the lighting of the scene and the distinction between background and user, in a simple and robust way. The ability to capture 3D data from a scene, which can be modelled like a point cloud, introduces the possibility of determining hand poses by means of depth clustering analysis. This analysis can be combined with some of the techniques used in RGB analysis, such as the use of morphological constraints [28] or dynamic skin colour segmentation [29]. The positions of the hands can also be obtained through more complex analysis, such as tracking the skeleton of the user [30]. In the latter case, there are several implementations of skeletal tracking available to the general public (OpenNI, Kinect SDK) that are sufficiently reliable for our proposed work. The proposed method involves several processing steps (Figure 3):

1. Detecting the skeleton of the user
2. Segmenting the area contiguous to the hand (rough segmentation)
3. Splitting hand points and noise from the extracted area (fine segmentation).

*Step 1: Using skeletal tracking* 

As the first step of hand segmentation, a skeleton tracking node provided by the manufacturer of the sensor (NiTE middleware with the OpenNI driver) is used. The tracking is carried out from the IR sensor and without using the RGB information. This is generally because IR sensors are less sensitive to changes of light in the scenario. Only interferences on the same wavelength could cause detection problems, but this is unlikely to occur in indoor environments and households which are lit by artificial light and their spectrum is away from infrared light. This has been empirically tested with different people and rooms. This basically means that the tracking system works properly with people of different races who may have varying skin colour and body shape. Besides, the tracking system can be used with people wearing any kind of clothes provided that the garments are not too voluminous. It is preferable for clothes to be as close fitting as possible. In our case, skeletal tracking is just for the upper body. The idea is to provide the least light-dependent and invasive system possible. In addition, the skeletal tracking can follow multiple users at the same time in the environment. The skeletal tracking can simultaneously follow multiple users in the scenario. This approach makes it easy to expand the capabilities of our system to the control of more than one target with a single sensor or to the collaborative control of the same target. The greatest disadvantages of the mechanism are the need for a starting pose for skeletal recognition and the need for the user to be positioned in front of the camera, with most of his or her body present in the field of view of the sensor.

In comparison with the official MS Kinect-SDK, the NiTE middleware offers greater flexibility for embedding it in an ultimate solution. Various binaries are provided that have been compiled for Windows and GNU/Linux systems, and in later versions, compatibility with ARM processors has been added, allowing the system to host applications on mobile devices and microcomputers. The maintainers of the most popular Linux distributions provide a ready-to-install package in the official repositories, and the maintainers of the Robot Operating System (ROS) middleware provide a package that is ready to add to the architecture of a standard ROS solution. Furthermore, in a comparison of the precision of the two skeletal tracking approaches, there are no noticeable differences between them in the normal use case [31].

*Step 2: Rough segmentation* 

**Figure 4:**
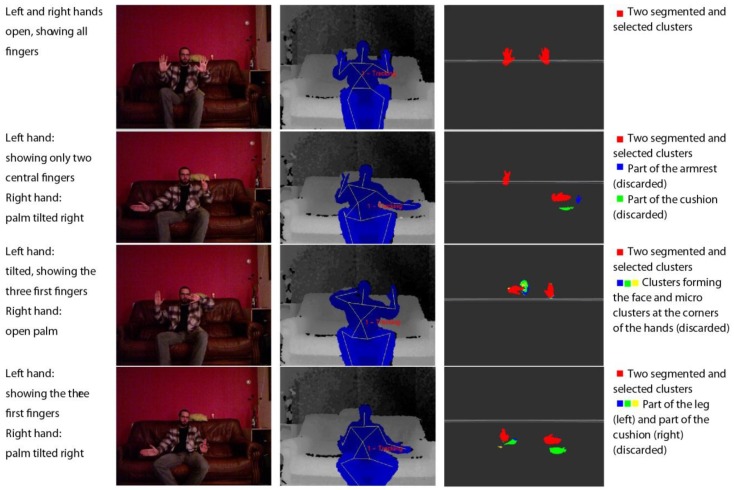
Representations of the detection of the human skeleton via the segmentation and filtering of a point cloud to detect the hands of a user sitting on a couch.

Once the approximate positions of the two hands are located, the next step is the extraction of the points in their surroundings. The cloud is ordered in a k-d tree. A k-d tree is a data structure that represents a binary tree and is used for ordering a group of k-dimensional components. In this case, we are organising a cloud of points by their spatial coordinates to enable an efficient range search on the neighbourhood of the detected centre of the hand (Figure 4).

For this step, a radius of interest of 24 cm around the centre is considered. This value is the sum, rounded up, of the average error on the hand position detection of a user in a sitting position (14.2 cm [31]) and half the average length of a male hand among members of the European ethnic group with the largest hand size (19.5 cm) [32].

*Step 3: Fine segmentation* 

**Figure 5:**
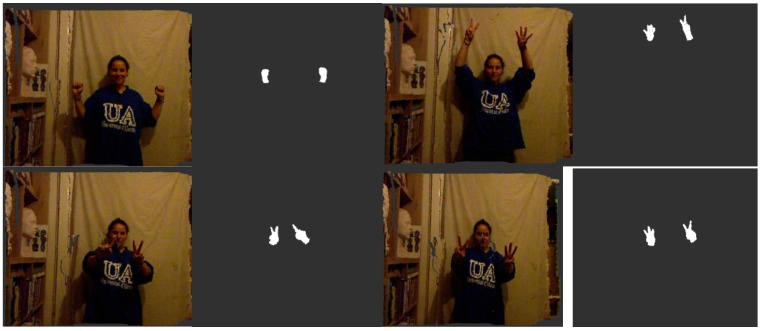
Visualisations of the original coloured point cloud and the final segmented hands of a user standing up.

The final processing step removes potential spurious elements that appear in the segmented area because of their proximity to the hands of the user. These elements could be objects in the scene as well as the clothes of the user or other parts of the body (e.g., chest, hair). Because the segmentation is initially centred on the palm of the hand, we assume that the largest continuous element in the extracted cloud will be the palm, the fingers attached to the palm and part of the arm. Then, the analysis is performed by defining a cluster as a set of points that are closer than 3 cm to another point in the cluster. This margin is needed because when the hand is located in a deep region of the scene, the density of points is lower and the segmentation could miss one or more fingers. Several constraints can be formulated to avoid false cluster identification, such as a minimum cluster size (to avoid falsely identifying noise as a signal when the hand is out of the scene) or a maximum cluster size (to avoid identifying one or more additional adjacent elements as part of the hand). Experimentally, we determined that reasonable values for these constraints are 100 points for the minimum size and 30,000 points for the maximum size (Figure 5).

#### 2.4.2. Gesture Recognition

Various approaches are used in the literature to address the problem of gesture classification. For example, classification can be performed based on pose estimation by imitating the skeletal tracking of the Kinect SDK [33,34] or through a combination of hand feature extraction (location, centroid, fingertips, silhouette) and machine learning (Hidden Markov Models [35], k-Nearest Neighbours [36], shape description [37]), combining different techniques in each step to construct the best system for the target application.

**Figure 6:**
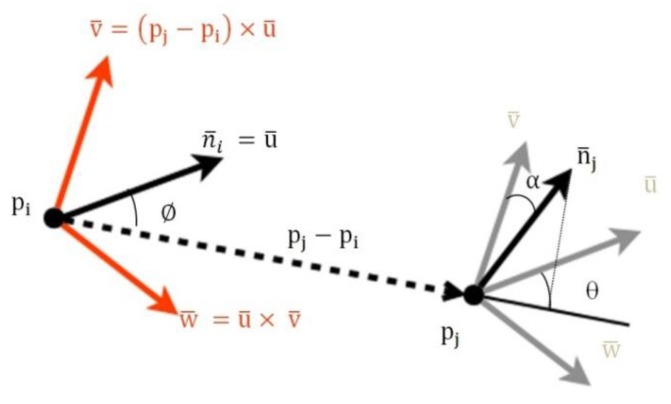
Geometric variation between two points based on the framework of Darboux transformations.

Once the hand is extracted, the process is split into two parts: training and detection. For both sub-processes, a descriptor of the segmentation result is computed. The descriptor used in our prototype is the Viewpoint Feature Histogram (VFH) [38]. The VFH encodes the differences in angle–pitch (α), roll (∅) and yaw (θ)–between the normal vector of the centroid p_i_ of the point cloud that represents the hand and any other part of the cloud p_j_ in the Darboux frame (Figure 6) [39].

The reference frame centred on $p_{i}$ is defined by:

(1) $$\left\{\bar{u},\bar{v},\bar{w}\right\}=\left\{{\bar{n}}_{i},{\bar{t}}_{i},{\bar{n}}_{i}\wedge {\bar{t}}_{i}\right\}$$

where

(2) $$\bar{u}={\bar{n}}_{i}$$

(3) $$\bar{v}={\bar{t}}_{i}=\bar{u}\wedge \frac{\left(p_{j}-p_{i}\right)}{d_{L2}}$$

(4) $$\bar{w}=\bar{u}\wedge \bar{v}={\bar{n}}_{i}\wedge {\bar{t}}_{i}$$

Then, the geometric variation between the two points can be expressed as the relative difference between the directions of their normal vectors ${\bar{n}}_{i}$ and ${\bar{n}}_{j}$, and it is calculated as follows:

(5) $$\alpha =\text{acos }(\bar{v}\cdot n_{j})$$

(6) $$\emptyset =\text{acos}\left(\bar{u}\cdot \frac{\left(p_{j}-p_{i}\right)}{d_{L2}}\right)$$

(7) $$\theta =\text{atan}\left(\bar{w}\cdot {\bar{n}}_{j},\bar{u}\cdot {\bar{n}}_{j}\right)$$

(8) $$d_{L2}=∥p_{j}-p_{i}{∥}_{L2}$$

where $d_{L2}$ represents the Euclidean distance between two points in the space.

**Figure 7:**
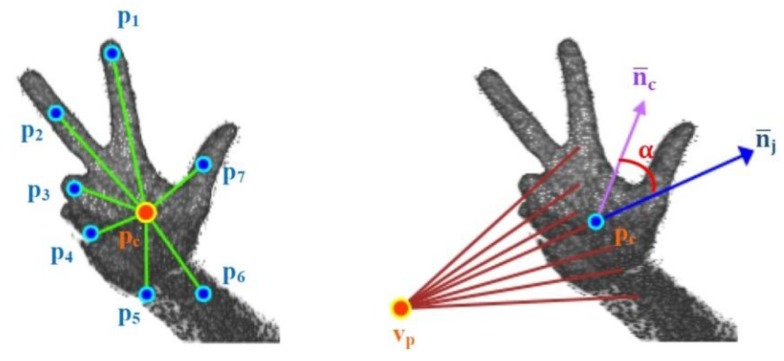
Viewpoint information of the VFH descriptor of a human hand.

**Figure 8:**
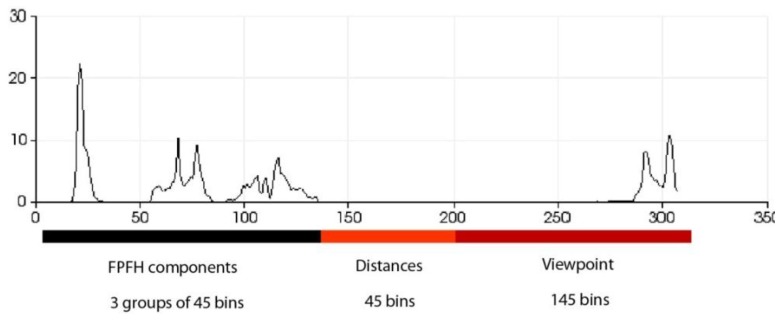
Description of the zones of the histogram.

This geometric variation is used to determine the geometric shape in a manner similar to that used other works, such as [40], but in this case, it is applied to hand gestures. Additionally, the VFH incorporates information to encode the direction of the point of view. For this reason, the VFH is suitable for recognising gestures as well as for processes that require identifying the pose of an object (Figure 7). The VFH descriptor for each point on the hand (Figure 7) is represented as a multi-dimensional histogram that accumulates the number of repetitions of a tuple $\left(\alpha ,\emptyset ,\theta ,d_{L2}\right)$ (Figure 8) where each component is normalized to 100. Furthermore, as in any one-dimensional histogram, it is necessary to split the data into a specific number of divisions. Each of these divisions represents a range of values of each element of the tuple and graphically indicates the number of occurrences belonging to each range of values (Figure 8). The descriptor normalizes its bins by the total number of points which represent the hand and also it normalizes the shape distribution component [41].

**Figure 9:**
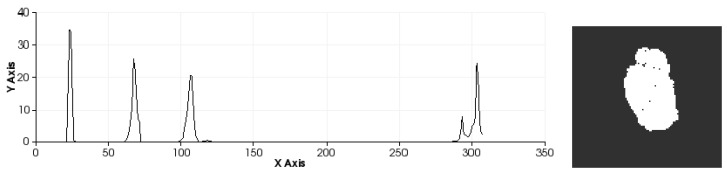
Sets of descriptors computed to recognise several left-hand gestures I. Descriptor in the left. Pose in the right.

**Figure 10:**
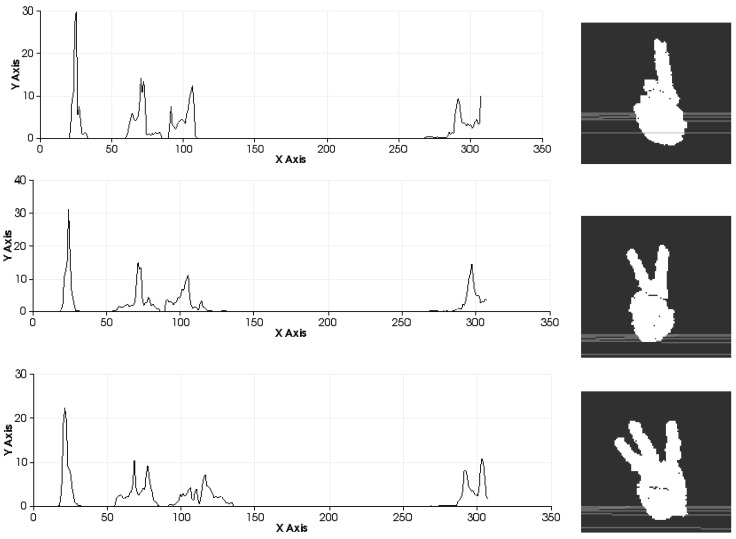

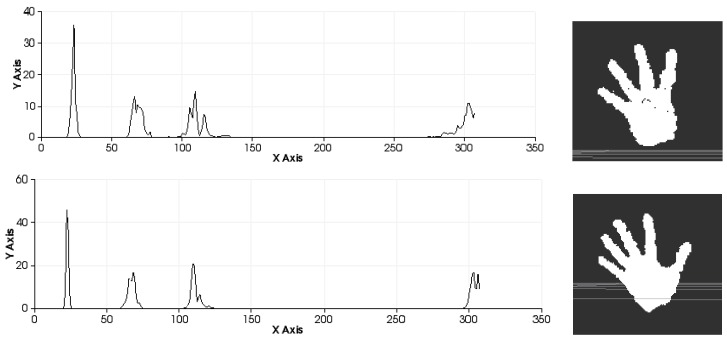
Sets of descriptors computed to recognise several left-hand gestures II. Descriptor in the left. Pose in the right.

**Figure 11:**
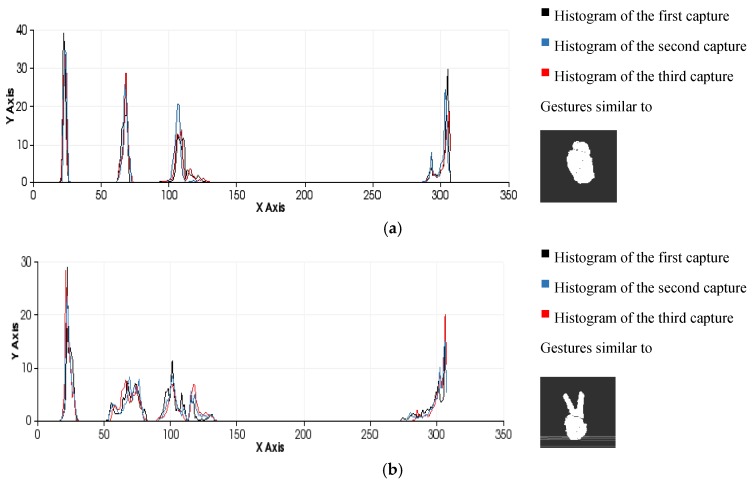
Examples of the gesture classification process performed by comparing histograms using the minimum Euclidean distance as a similarity metric. (**a**) Gesture 0 (test 1=31,test 2=25, test 3=32); (**b**) Gesture 2 (test 1=27,test 2=41,test 3=33).

This approach yields a descriptor that is well suited to our purpose because it is global, rapid to compute, independent of scale and dependent on pose (Figure 9 and Figure 10). This last feature will allow us to decide at the time of training whether two different poses should be considered to be the same gesture or different gestures (Figure 11). Once the possible gestures are defined, several descriptors of the different frames of each gesture are stored. The method that is applied to match the current gesture with one of the trained gestures is similar to that used by Rusu *et al*. [31]. All the histograms that describe a gesture are regarded as 308-dimensional points (one dimension per bin) and are placed in a point cloud. Afterwards, the incoming gesture is placed on the cloud, and the 10 points (histograms of the analysed gestures) that are nearest to it are located. Because these points may be frames of the same gesture or of different gestures, the matching gesture is determined via weighted voting, based on the distances between the histograms.

**Figure 12:**
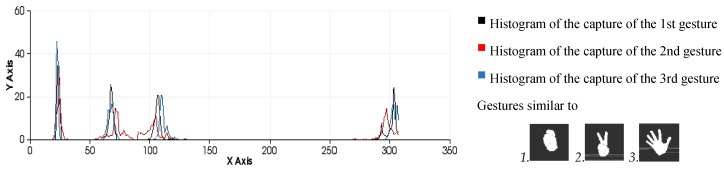
Similarity measures computed during the classification process among three different human-hand gestures: gesture 0, gesture 2 and gesture 5 (test 1=81,test 2=45,test 3=87).

The effectiveness of the entire process (segmentation and recognition) is highly dependent on the number of different gestures to be distinguished and the differences between the gestures due to the variance in the morphological features and poses (Figure 12). It is possible to perform pose-invariant recognition while training the algorithm, grouping the same figure in different poses as the same gesture and increasing the number of frames captured per gesture. In general, we can affirm that for a given number of gestures to be distinguished, a higher number of frames per gesture in the database will result in higher precision.

It is possible to distinguish several differences between histograms of each example (Figure 9 and Figure 10) with regard to the first part of the histogram representing the FPFH components encoded as a set of angle variations, such as pitch (α), roll (∅) and yaw (θ) (Figure 8). All of these are calculated using the same number of repetitions per value. Figure 9 shows the representation of 0 fingers by means of close hand gesture, and Figure 10 shows five samples of the most common representations from 1 to 5 that can be represented with the hand gestures by extending fingers. A comparison between the first and fifth samples of Figure 10, regarding the first part of the histograms, shows the changes in the dispersion of angle variations. Thus, the first sample shows that there are more bins with no zero values and the histogram has a greater distribution of angular values. That is to say, the first sample has more dispersion than the fourth or fifth sample. But also, the fifth sample shows that the angular values are more concentrated around three bins than in the remaining samples in Figure 10. To summarise, that concentration occurs for different values in each histogram, resulting in sharp concentrations on an entropic background. 

Comparison of all the figures denotes that the differences on the last part of histogram, which represents the viewpoint, are mainly caused by the changes in the position of the hand and the camera in the whole training process. Note that they are less related to the gesture morphology (*i.e*., with the hand pattern shape which is being reproduced by the user). These variations can be observed in Figure 12 which represents the overlap of three gestures.

#### 2.4.3. Generating Commands via the Combined Interpretation of the Movements and Gestures of Two Human Hands

A scripting module has been built for the tele-control of Charlie using its socket API. Each gesture associated with each hand commands a state change. With the left hand, we select the orientation of the wheels (palm to the left, pointed left; palm to the right, pointed right; closed hand, pointed straight). With the right hand, we command the rotation of the wheels (open palm, forward; closed hand with the thumb extended thumb, backward; closed hand, brake). The relative spatial position of the hands in the scene is used to control the orientation of the camera on the robot. Separating the hands will cause the angle of the camera to rotate upwards, and bringing the hands closer together will cause it to rotate downwards. In addition to this functionality, the absolute positions of the hands in the scene are considered to make the system more usable for a person with reduced mobility. For a user sitting in a chair, the action of rotating the camera will be performed if the hands are above the neck. The gestures will be interpreted as orientation and rotation commands if the hands are positioned between the neck and the hips. Positions below the hips will be regarded as resting positions.

## 3. Experiments and Results

### 3.1. Experiment 1: Programming and Controlling the Robot

**Figure 13:**
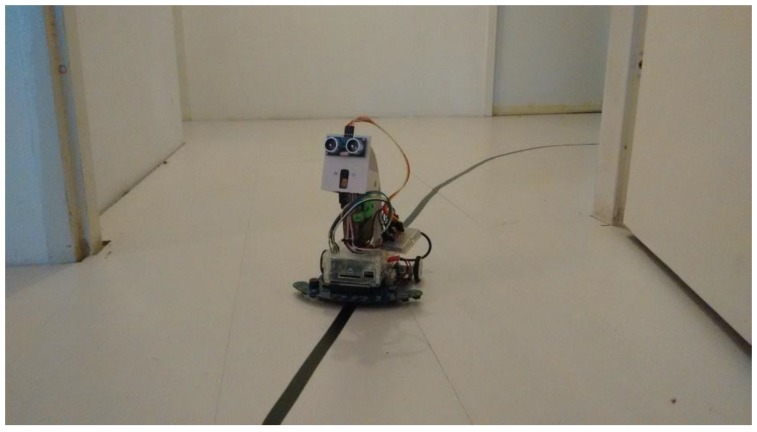
Our low-cost robot controlled by our human-hand gesture interface.

Using the scripting API, it is possible to create behaviours for the robot. In the experiment described below, the upper row of 12 CNY70 reflective optical sensors with transistor output is used to perform a line-following movement strategy (Figure 13).

The goal is to implement a simple line-following behaviour by obtaining the position of the first sensor (from the left) to detect a line. Then, this initial value of between 0 and 11 (x) is rescaled to the range 0–255 and sent to each motor in reverse. The applied formula is as follows:

(9) $$\begin{array}{c} speedLeft=\frac{255}{11}x\\ speedRight=255-speedLeft \end{array}$$

For example, if a line is detected by the first sensor (from the left), the resulting value of the find function will be 0. Then, the robot will need to perform a strong turn to the left, which requires the speed of the left motor to be 0 and that of the right motor to be 255. These actions are repeated in a loop (read the sensors, calculate the speed of the motors and send the movement command to the Arduino board) as long as the read-out of the sensors continues to indicate a black line; when this is no longer true, the command to stop the motors is executed. For this procedure, three commands of the low-level API are used:

- executeReadCommand(14): Returns a string of length 12 that contains the read value of each sensor in the row. For example, the string “100000000000” indicates that a line has been detected by the top left sensor.
- executeCommand(5,speedLeft,speedRight): Execute the movement corresponding to the specified speed of each motor, from 0 to 255.
- executeCommand(0): Stop the movement of the motors.

We can store this command by sending the WebSocket server a message in which the first byte is 50, the second byte is the ID of this script (from 0 to 255), and the remaining bytes contain the code itself. Then, for execution, the command 50 followed by the assigned ID must be sent to the WebSocket server.

### 3.2. Experiment 2: Results of the Detection of Hand-Human and Gesture Recognition

**Figure 14:**
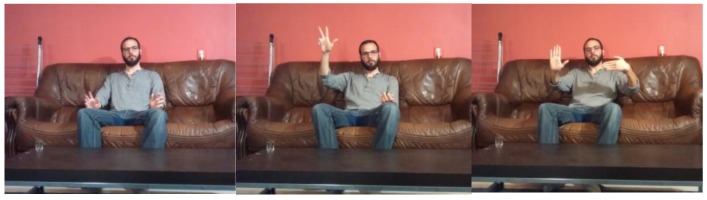
A user at rest, issuing commands with the right hand and issuing commands with both hands.

As a result of the aforementioned gesture recognition parameters, the effectiveness of the recognition results is highly variable. To illustrate our proposed approach, we considered three and six different gestures per hand. The system was trained using a database of 250 samples per gesture in both experiments (Table 3 and Table 4). All gestures were captured by the Kinect sensor in real time from different environments and different users (examples are shown in Figure 4, Figure 5 and Figure 14). In Table 3 and Table 4, the columns represent the gesture models, and the rows represent 600 samples of unknown gestures that were captured by the Kinect for classification.

**Table 3:** Confusion matrix computed during the recognition process for the identification of three simple gestures of the right hand, with 200 samples per gesture.

	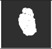	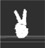	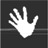
	Recognised Gesture 0	Recognised Gesture 2	Recognised Gesture 5
Input Gesture 0	200	0	0
Input Gesture 2	3	195	2
Input Gesture 5	15	6	179

**Table 4:** Confusion matrix computed during the recognition process for the identification of six gestures of the right hand, with 200 samples per gesture.

	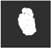	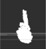	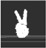	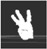	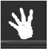	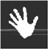
	Recognised Gesture 0	Recognised Gesture 1	Recognised Gesture 2	Recognised Gesture 3	Recognised Gesture 4	Recognised Gesture 5
Input Gesture 0	177	3	0	2	18	0
Input Gesture 1	4	177	10	7	2	0
Input Gesture 2	1	21	133	32	8	5
Input Gesture 3	0	0	0	184	16	0
Input Gesture 4	0	0	0	8	176	16
Input Gesture 5	23	0	5	8	36	128

The hit rate decreases when the number of gestures in the database is incremented, as shown in Table 4 which includes a further three gestures, more than in Table 3. The success rate depends on the morphological differences between gestures. As a result, two similar gestures could often be mismatched when the database is small but when it is increased, they could also be more likely to be matched. That is to say, the probability of the gesture recognition process decreases with the size of the database and number of gestures previously registered. For this reason, in our system, the use of more than four gestures/hand could easily cause confusion. To attenuate this fact without sacrificing the robustness in detection, our system seeks to work with four gestures of both hands, simultaneously. Therefore, two small sets of several different gestures are registered in our database (eight gestures, four for each hand). This set is used in the experiment shown in Section 3.4. Those gestures are the representations of zero, one, three and five fingers. However, it is very important to consider the fact that our system is able to work with combinations of sequences of three concatenated gestures for each hand. Therefore, combinations of three elements can be used to identify action commands. Consequently, the system works by forming a State Machine (SM) with multiple actions where the input is a sequence of gestures combining both hands, and the output is a reliable transition between states. Therefore, the difference of a single hand gesture allows the system to be associated with different actions.

The choice of gestures is arbitrary and is not associated with the numerical value shown. It is associated with actions and commands for the robot. The descriptor is not invariant to poses, thus a gesture is labelled as different even though it was obtained from different hand orientations but also with the same visible fingers (*i.e*., same fingers pointing up or down).

An important aspect of evaluating the suitability of our proposed method is the reaction time required to detect a gesture. The following measurements, presented in units of milliseconds, were executed under full-stack conditions; in other words, all processes were running at 100% system load. The computer used for this purpose was a Lenovo T520 (Intel Core i5 2520 M @ 2.5 GHz, 6 GB of RAM DDR3, Intel HD3000). The durations were measured for 100 instances of detection (Table 5 and Table 6).

**Table 5:** Times required for different steps of the hand segmentation process.

	Max (ms)	Average (ms)	Min (ms)
Rough segmentation	242	203	165
Fine segmentation without noise	101	62	37
Overall process (segmentation, neighbourhood search and data conversions between steps)	1222	513	383

**Table 6:** Times required for different steps of the gesture analysis and classification process.

	Max (ms)	Average (ms)	Min (ms)
VFH analysis	177	120	37
k-d tree search with 750 elements	7	4.4	2
Overall process (analysis, search and data conversions between steps)	533	196	71

### 3.3. Experiment 3: Controlling the Robot with Human-Hand Gestures in a Domestic Environment

Regarding the hand gesture recognition software, an ROS node that monitors the data sent from the skeleton tracking and gesture detection modules was implemented. This node represents a coupling between the full detection chain and the hardware of the robot, transforming the currently detected scene into a command. The position of the hand relative to other elements of the body (e.g., head, shoulder, hip) is used to determine whether the user is re-posing his or her hands or activating commands. The same gesture can be associated with different commands depending on the relative height of the hands. Once the pairs of gestures and positions are associated, we add a third element to the tuple, namely, the desired action of the robot. A number that identifies this action is sent via the WebSocket to the robot using its specialized API.

On the part of the robot, two API commands are used. Scripts must first be pre-programmed using JavaScript; the system then stores the various specified behaviours in memory using the store script command. Once they are stored, they can be executed using the load and execute commands. Therefore, for this experiment, the robot received these two types of commands via the WebSocket; only the parameter that indicates the ID of each script, which takes values in the range 0-255, is modified among different instances of such commands to indicate the slot in which the desired script is stored. 

The error was then measured as the deviation between the central sensor (Figure 1) and the read sensor. Because there are 12 sensors (with labels x ranging between 0 and 11, where the central sensors are numbered 5 and 6), the formula for this deviation error is:

(10) ε=|5.5−x|

where *x* is the label of the read sensor; thus, the error represents the position of this sensor in relation to the central sensor.

**Figure 15:**
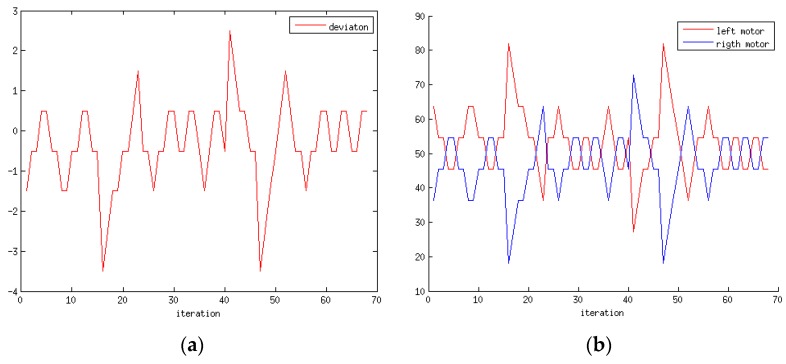
(**a**) Deviation of the line detected by the robot’s sensors; (**b**) Speed applied to each motor by the closed-loop controller to correct the path.

Suppose that a command is sent to the robot to move to a certain location in the environment. It should be noted that the objective of this work is not the localisation of the robot or the mapping of the environment (SLAM) along the navigation paths. The primary goal is to develop a low-cost robot with a motorised camera such that the movements of the robot and camera can both be controlled by a gesture interface based on visual perception for the tasks of supervising and monitoring a household environment. The general movement of the robot is controlled by an open-loop controller based on 3D data that are acquired by an external RGBD sensor, and the path of the robot is controlled by a closed-loop controller based on data from sensors that are mounted on the robot; they are used, for example, to allow the robot to follow a black line on the floor to move from one room to another. Figure 15 illustrates the behaviour of the low-cost robot following a path between two rooms as commanded by a human user via a hand gesture. First, the gesture is recognised, and the corresponding command is sent to the robot; then, the robot activates and moves to achieve its objective. Afterwards, the movement of the robot is controlled by its sensors such that it maintains a certain distance with respect to the desired path (curved or straight). Figure 15a shows the deviation from the desired path. In this case, sensors 5 and 6 (Figure 1) exhibit oscillations due to measurement errors and the robot’s velocity. For both straight lines and curves, the trajectories tend to exhibit an oscillation of approximately ε = 0.5. Although there are initial peaks with a deviation of ε = 3.5 (38.5%), these occurrences do not pose a problem for the robot in tracking its path to achieve its target. In general, the algorithm applies more power to the motor that is opposite to the position of the detected line, as shown in Figure 15b, to correct the position in relation to the desired path.

### 3.4. Experiment 4: Behaviour of the System in a Complete Use Case 

**Figure 16:**
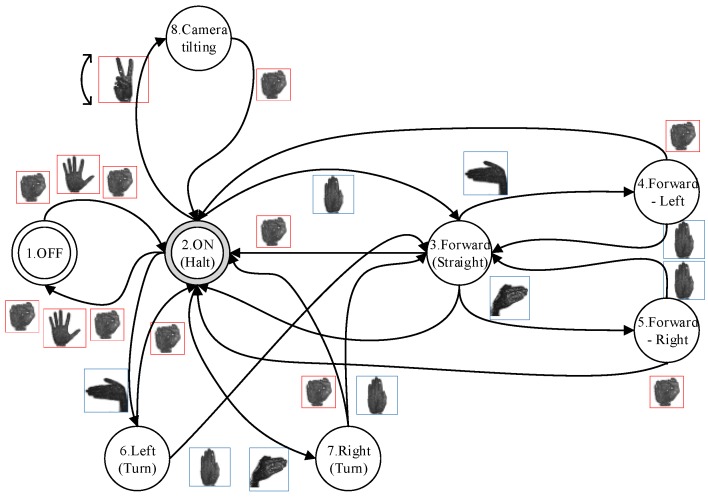
Navigation through a finite-state machine by means of triggering gestures.

**Figure 17:**
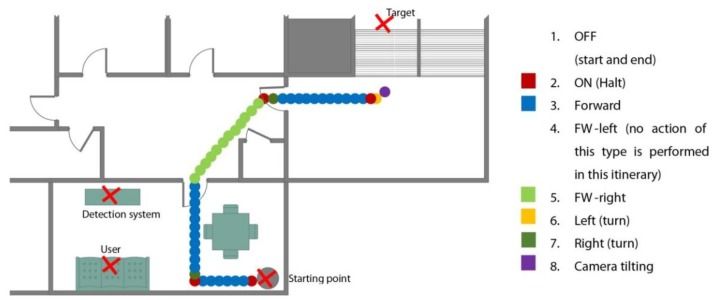
Driven itinerary throughout the house, with the state detected at each point.

This experiment describes an instance of the use of the full system (Figure 16) in the residence of a dependent person (Figure 17). 

The proposed system can recognize four gestures and the actions can be composed of sequences of one, two or three gestures (which are represented by zero, one, three and five fingers). The mathematical combination of these items provides up to 84 sequences for allocation of robot orders or actions. That is to say, there are four combinations of one gesture, 16 of two gestures and 64 of three gestures that can be generated from this set of gestures. However, the implemented system was only tested with the restriction of three instead of four gestures (zero as a closed hand, five as an open palm with separated fingers, and two representing the victory sign). In this case, the number of actions is reduced to 39. In command words, the set of 39 actions is given by a sequence of k not necessarily distinct gestures, where k can take the value one, two or three, and where the command is not taken into account. This means three combinations of one gesture, nine of two gestures and 27 of three gestures. Moreover, the choice of gesture was made with the intention of making each robot action a natural one. For example, to go forward, it uses the gesture of the right hand with fingers up.

Furthermore, our system works like a SM where each combination of gestures could define one action at a time. This means that the robot actions depend on the current state and the sequence of gestures used by the human operator. The machine can change from one state to several different states with the same combination if the current state is different. Therefore, the transitions are defined by a list of previous states and the triggering condition defined by the gestures.

Figure 16 shows an example of actions associated with states and sequence of gestures. Thus, the SM indicates that a sequence of three gestures on the left hand is needed in order to go from OFF status to ON status. Moreover, a sequence of two gestures on the right hand is used to go from ON status to Forward-Left status. In this case, the gestures are with the left hand opened (ON to Forward, Forward to Forward-Left). Also, a closed hand on the same hand allows the robot to pass from any status to Halt mode (ON). In the same way, other additional actions have been associated with the set of gestures, such as the tilt camera movement which is controlled by the variation measure on the height of the left hand, when it represents the victory sign. This variation is mapped according to the amount of degrees of the camera servomotor required to rotate it.

The user is resting on a couch when he hears an audio notification from a sensor at the main entrance of the home indicating someone’s arrival. The user wishes to determine who is coming in and, for that purpose, performs the following sequence of movements: (1) places his right hand in the allowed detection area (between the hip and the neck); (2) performs the sequence for initiating gesture detection, which is composed of three consecutive gestures; (3) directs the robot (by performing the necessary gestures) to the main entrance; (4) tilts the camera to focus on the face of the newly arrived person; (5) moves the robot out of the way; and (6) performs the sequence for terminating gesture detection (the same sequence as in step 2). 

Some gestures consist of more than one pose. No constraint is placed on the time allowed to perform each of the poses of a gesture. The gesture used in step 4 depends on the relative variations in the height of the position of the hand: 2 cm of variation (up or down from the starting height) will be translated into 5° of rotation of the axis of the camera. The range of the servomotor that controls the inclination is limited to 135°, and thus, the maximal distance that must be moved between hand positions is 54 cm. Each state is associated with a high-level API command for the robot, with the exception of the Halt state. This latter is executed by the machine that is connected to the Kinect (Table 7) because it is related to the initiation or termination of operation. 

**Table 7:** Charlie API commands associated with different states:

	Command	Parameter 1	Parameter 2	Command Description
**1. OFF**	0			Stop Motors
**2. ON (Halt)**	(managed by the machine connected to the Kinect)			
**3. Forward**	1	191		Move forward (both motors at 75% speed)
**4. Forward-Left**	5	63	191	Left motor 25% Right motor 75%
**5. Forward-Right**	5	191	63	Left motor 75% Right motor 25%
**6. Turn Left**	6			Edge-rotation to the left
**7. Turn Right**	7			Edge-rotation to the right
**8. Camera Tilting**	35	x		Sets the inclination of the camera

**Figure 18:**
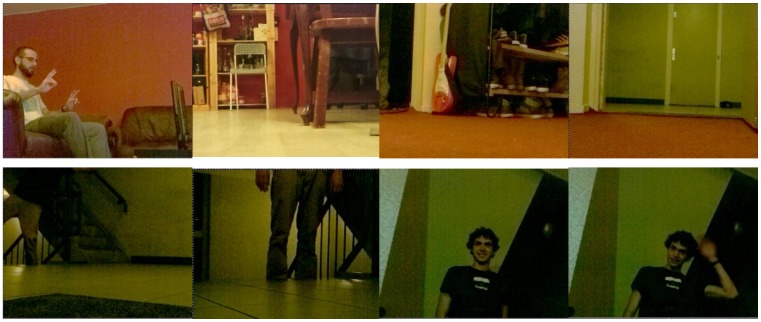
Frames captured by the robot’s camera along the itinerary from the base station to the target.

As seen, the robot is not capable merely of reproducing marked trajectories; it can also be programmed to perform actions in a different manner through multi-pose gesture recognition. Via the wireless network of the house, it is possible to access the video stream from the camera of the robot using a simple web browser, providing the user with first-person feedback (Figure 18). Increasing the complexity of the gesture detection, by including a finite-state machine in between, improves the rejection of faulty detections and makes the output more reliable (Table 8). To filter out any remaining errors prior to changing the state of the machine, a vote-based evaluation of three incoming gestures is performed, and the gesture with the most identified occurrences is accepted as the intended one.

**Figure 19:**
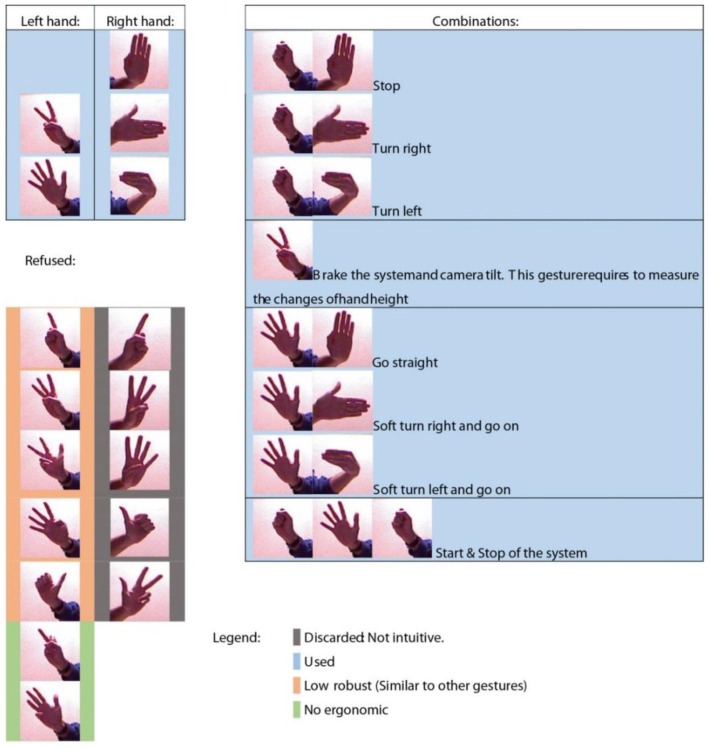
Gestures considered for robot control in our experiments.

**Figure 20:**
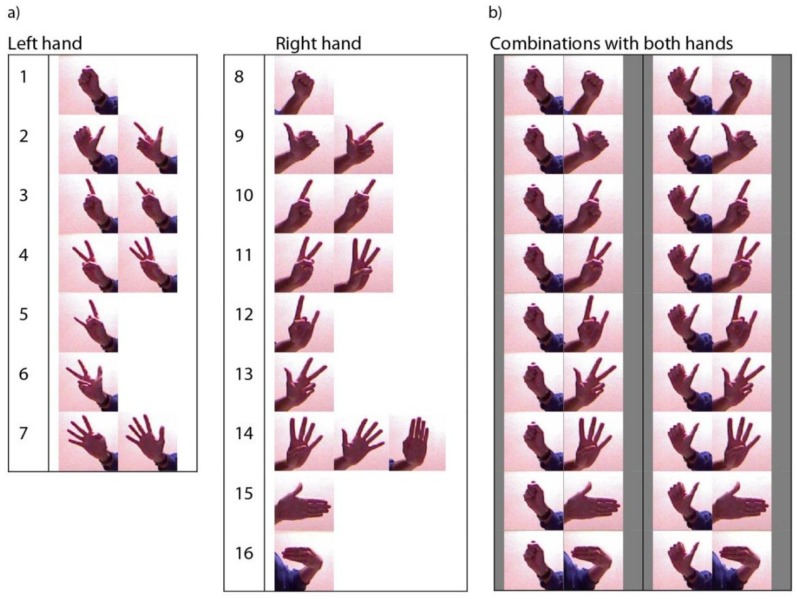
Full set of gestures that the recognition descriptor is able to identify correctly and are considered for handling the robot. (**a**) With just one, left or right hand; (**b**) Sequence of two gestures with both hands.

Figure 19 shows the set of the used gestures which were chosen because they are easily imitable by anyone (ergonomic) and provide a good recognition result. In addition, it shows the refused gestures which were not considered due to several reasons such as difficult imitation, excessive similarity with other gestures (success rate of the descriptor in the recognition process decreases significantly) or the gesture is not intuitive; *i.e*., it would be difficult for the user to remember the action according to shape and position. Figure 20 shows the full set of gestures that the recognition descriptor is able to correctly identify and are considered for the handling of the robot. It is important to note that the grouped gestures which have been labelled with the same number cannot be used together in our system because the recognition descriptor does not provide a suitable difference between both signatures. Also, the figure illustrates the different combinations chosen for both hands. The criterion was to choose the most robust gesture for the left hand and the most intuitive one for the right hand.

**Table 8:** Accuracy of the detection of gesture sequences to reach the state needed to determine the path of the robot in Experiment 4 and in artificial tests of the navigation between states (100 trials per action).

	Involved States (1)	Times Detected (2)	Times Missed (3)	Times Missed after 3-Step Voting Evaluation (4)	Ratio of Correct Detection in Artificial Tests after 3-Step Voting Evaluation (6)
Unlocking and locking	1, 2	2	1	0	100%
Moving forward	2, 3, 4, 5	32	6	1	92%
Moving left	2, 6	3	0	0	97%
Moving right	2, 7	8	1	0	95%
Moving forward-right	3, 5	14	3	0	95%
Camera tilting	2, 8	6	0	0	100%
Halt	All to 2	13	0	0	98%

## 4. Discussion and Conclusions

This paper presented a low-cost robotic system to assist people with monitoring tasks in home environments. The robotic system is managed by means of a human interface that recognises gestures from both hands. Our human interface consists of a low-cost RGBD sensor, such as the Kinect, and a set of algorithms to detect the locations of human hands based on the skeleton of the user’s body and to recognise their poses and orientations using a 3D descriptor of surfaces. This component of the system functions correctly regardless of the characteristics of the environment and the human operator. 

The use of a low-cost robot to perform the proposed tasks is a new approach to reducing the cost of the high-level robotisation of tasks in the home. This approach introduces the possibility of controlling the system with human gestures and provides the user with feedback from the environment via a motorised camera. The primary advantage of this low-cost approach is the ease of replacement of any damaged component; as a trade-off, the designed prototype is not highly robust against unexpected problems that may arise in its operating space.

The robotic system has been programmed to be used as a basis for surveillance activities in the home. It moves through the house and uses a motorised camera, which is also remotely operated by gestures. In this way, dependent individuals can require fewer other people for assistance over time. The robot is intended to assist the disabled, the elderly or people with mobility problems in tasks that involve physical actions, such as getting up and moving from one place to another inside the home to observe what is occurring in those locations.

The results of the experiments indicate that the proposed human interface based on hand recognition achieves high levels of accuracy for the interpretation of gestures (greater than 86%). Although the experiments also indicate the occurrence of false positives in the recognition process, the system always processes in real time and allows the human operator to repeat a gesture three times to ensure that the interpretation is robust and to prevent unwanted or erroneous task commands being issued to the robot. This repetition concept is based on the High Dynamic Range (HDR) mode of certain cameras, in which each gesture is automatically captured three times, and its intent is to improve the range used to register the 3D point cloud that represents the hand. Additionally, a runtime study revealed that the mean runtime of the entire recognition process is 320 ms. This process includes the image acquisition, feature extraction, 3D descriptor computation and matching between the test view and the model database. Two disadvantages are that the system requires training and that a larger number of possible gestures increase the runtime and decrease the level of accuracy, thereby reducing the success rate. However, a considerable advantage is the possibility of working with two hands and the implementation as a State Machine (SM) and consequently, it is possible to associate more robot commands or actions than gestures because the actions depend on the current status of the SM as well as the sequence of gestures, and not just the latter.

Another important aspect is avoidance of obstacles; the distance sensor mounted on the robot is used for this purpose. If it detects an obstacle, the robot stops and waits for new commands from the user, who controls the robot movements with his gestures using the view captured by the webcam mounted on the robot. In summary, the designed system offers a new approach to the execution of tasks in the home. Currently, the system is being improved through the development of an easy programming method for defining the tasks and behaviours of the robot.
